# Porous Oxygen-Doped g-C_3_N_4_ with the Different Precursors for Excellent Photocatalytic Activities under Visible Light

**DOI:** 10.3390/ma15041391

**Published:** 2022-02-14

**Authors:** Jiajing Zhang, Yongjie Zheng, Heshan Zheng, Tao Jing, Yunpeng Zhao, Jingzhi Tian

**Affiliations:** School of Chemistry and Chemical Engineering, Qiqihar University, Qiqihar 161006, China; zhangjiajing_1994@163.com (J.Z.); zhengheshan001@163.com (H.Z.); jtkr@163.com (T.J.); zhyypp@163.com (Y.Z.)

**Keywords:** copolymerization, g-C_3_N_4_, oxygen-doped, visible light degradation, photocatalysis

## Abstract

Antibiotic contamination has received widespread attention globally. In this work, the oxygen-doped porous graphite carbonitride (g-C_3_N_4_) was prepared with urea and ammonium oxalate (CNUC) or urea and glycine (CNUG) as precursors by thermal polymerization. Using bisphenol A (BPA) as a probe and CNUC or CNUG as photocatalysts, the removal performance test was carried out. Meanwhile, all prepared photocatalysts were characterized by XRD, FT-IR, SEM, TEM, XPS, UV-Vis DRS, PL and EIS. Under visible light irradiation, both CNUC and CNUG exhibited about seven and five times greater photocatalytic activity than that of pure g-C_3_N_4_, respectively. The radical capture experiments verified that superoxide radicals (•O_2_^−^) and holes (h^+^) were the main active species in the photocatalytic degradation of BPA by CNUC, and the possible photocatalytic mechanism of CNUC was proposed. In addition, all these results indicate that CNUC catalyst can effectually inhibit the photocorrosion and keep superior stability. The proposed technique provides a prospective approach to develop nonmetal-modified photocatalysts for future applications.

## 1. Introduction

Bisphenol A (BPA), also named diphenol-based propane, is a low-toxic compound and an important raw material for the production of polycarbonate, epoxy resin, phenolic resin, some polysulfones, and some special materials [1]. Because of its light weight, transparency, and toughness, BPA is widely used in manufacturing notebooks, mobile phones, baby bottles, and other food and beverage containers [2]. However, experiments have proved that BPA has an endocrine disruptor effect, which can be transferred to the environment through plastics, and it is increasingly detected in water environments, posing a serious threat to the ecosystem [3]. At present, a variety of treatment technologies have been studied, such as chemical reactions [4], biodegradation [5], and physical absorption [6]. The removal of BPA could reduce harm to the human body and could reduce water pollution. Therefore, removing BPA is the focus of current research. Among various methods, photocatalytic technology is the most likely and effective method to remove phenolic pollutants in water [7]. Photocatalytic technology converts low-density solar energy into high-density chemical energy and can directly use solar energy to degrade and mineralize various organic pollutants in water and air. It has the advantages of mild reaction conditions, no pollution, and low cost [8], making it an ideal piece of technology for environmental pollution control and clean energy production. However, the current problem with photocatalysts is the rapid recombination of photogenerated electron–hole pairs and the narrow photoresponse range of the photocatalyst. [9]. Therefore, the development of high-efficiency photocatalysts is still one of the focuses and core issues of photocatalysis scientific research at present, and it will likely still be in the future.

Graphite carbonitride (g-C_3_N_4_) has gradually become a research hotspot since its discovery in 2009 as an n-type nonmetallic polymer semiconductor for the photocatalytic production of hydrogen [10]. The uniqueness of its semiconductor energy band structure and favorable chemical stability has been extensively used in the fields of photocatalytic hydrogen production [11], conversion of environmental pollutants [12], and reduction of carbon dioxide [13], and has always attracted people’s attention. As a photocatalytic material, it has many advantages: (1) Carbon nitride has a unique layered structure, which can provide more active reaction centers and play a role in many application fields. It can also improve or strengthen the functionality of the host material, endowing it more excellent performance. (2) Carbon nitride itself is stable and not only has the advantages of organic polymer molecules and layered structure but also has great doping changes. In addition, it has a simple composition, no metal elements and does not cause secondary pollution to the environment during use. (3) The bandgap energy of carbon nitride materials is higher than those of conventional semiconductors. Smaller band gap materials help to better absorb and utilize sunlight and have good application prospects in many fields, such as environmental purification [14]. However, the currently synthesized g-C_3_N_4_ still has shortcomings, such as small specific surface area and too fast photo-generated carrier recombination, resulting in low photocatalytic activity.

In recent years, researchers have been committed to studying the modification of g-C_3_N_4_ to improve the photocatalytic efficiency of g-C_3_N_4_. The modification methods include element doping, the formation of heterostructures with other semiconductors, and the construction of micro-morphology [15]. In these modification methods, the material for constructing the porous structure could increase the specific surface area of the catalyst and increase the carrier diffusion channel, thereby improving the light trapping capacity of the catalyst, accelerating the mass transfer process, leading to destruction and having more reactive sites. [16]. Besides, the nonmetallic elements used for doping include C, N, O, P, B, S, among others, which can further effectively improve g-C_3_N_4_ by changing the chemical properties of g-C_3_N_4_, narrowing the bandgap, and adjusting the electronic structure [17]. Regarding photocatalytic performance, Chen [18] had reported that porous oxygen-doped graphitic carbon nitride nanosheets (xSO-g-C_3_N_4_) were prepared using urea and sodium oleate via thermal copolymerization. The change in electronic structure promotes the formation of the intermediate band gap, resulting in xSO-g-C_3_N_4_ having good photocatalytic activity. Similarly, Zhu [19] prepared nitrogen-doped g-C_3_N_4_ (NCN) with a highly narrow bandgap and implemented it for the photodegradation of phenols. NCN (2:2) showed about two times higher photodegradation efficiency and three times higher rate permanent than the pristine g-C_3_N_4_. However, some disadvantages may exist in S or P doping, such as the generation of by-products and the dissolution of heteroatoms. In addition, during the current element doping to prepare porous g-C_3_N_4_ materials, in the preparation process, most of the templates (SiO_2_ [20] and SBA-15 [21], etc.), strong acids and alkalis were used for post-processing (H_2_O_2_ [22] and HNO_3_ [23], etc.). Therefore, it is necessary to develop a simple and green method to overcome the above shortcomings and apply them in practice.

Herein, two oxygen-doped porous g-C_3_N_4_ photocatalysts were successfully prepared by the thermal copolymerization of two different precursors. It is worth noting that, compared with pure g-C_3_N_4_, the two photocatalysts had significantly improved photodegradation efficiency of bisphenol A (BPA) and had excellent stability under visible light irradiation. Through the characterization of CNUC and CNUG’s morphology, chemical composition, light absorption and photoelectric properties, the reasons for their interactive photocatalytic effect were explained. The as-prepared catalyst had a large surface area and an extended light absorption region. Meanwhile, the two catalysts were compared to determine the optimal preparation method. Finally, the intermediates of the catalyst to degrade BPA were explored by LC-MS, and the possible degradation mechanism of the catalyst was proposed.

## 2. Experimental Section

### 2.1. Materials and Reagents

All chemical reagents and materials were purchased and used without further purification. Urea ((NH_2_)_2_CO) was bought from Profile (Wuxi, China). Ammonium oxalate ((NH_4_)_2_C_2_O_4_) and glycine (C_2_H_5_NO_2_) were purchased from Aladdin (Shanghai, China). Ultrapure water (>18.2 MΩ) was used in this study.

### 2.2. Preparation

The detailed preparation processes were provided in the Supplementary Materials section.

### 2.3. Characterization

The crystal structure of the prepared samples was characterized by X-ray diffraction (XRD), and the diffraction system was German BRUKER-AXS D8(Beijing Beishide Instrument Co., Ltd., Beijing, China). The chemical composition and element bonding of the prepared samples were analyzed by X-ray photoelectron spectroscopy (XPS) (Beijing Oubotong Optical Technology Co., Ltd., Beijing, China), and the XPS spectrum was recorded on the ESCALAB250Xi system equipped with a monochromatic Al Kα X-ray source. The chemical composition of the sample and the nature of the chemical bond are characterized by Fourier Transform Infrared (FT-IR) Spectrometer, Nicolet380 (Beijing Beishide Instrument Co., Ltd., Beijing, China.). The surface and micro morphology of the sample were characterized by using a scanning electron microscope (SEM) (S-3400) and a transmission electron microscope (TEM) (Tecnai) of Hitachi, Japan. The optical properties of the material were characterized by a diffuse reflectance spectrophotometer (UV-vis DRS, UV-3900) manufactured by Beijing General Analysis Co., Ltd., Beijing, China. The electrochemical properties of the material were recorded using the CHI 660B electrochemical system (Beijing Pofila Co., Ltd., Beijing, China) to record the impedance spectroscopy (EIS) Nyquist plot.

### 2.4. Photocatalytic Degradation of BPA

BPA was used as the target pollutant to evaluate the photocatalytic activity of the catalyst. We used a wavelength above 420 nm and a 500 W xenon lamp as a light source. First, we dispersed 50 mg of photocatalyst in 50 mL of BPA aqueous solution (20 mg/L), and magnetically stirred for 10 min in the dark to ensure that the adsorption–desorption equilibrium was reached before irradiation. During the reaction, stirring was continued to ensure that the mixture was in suspension. Every 10 min, we used a pipette to take out 5 mL of the solution and filter it with a 0.22 μm microporous membrane to remove the catalyst. Finally, high-performance liquid chromatography (HPLC) was used to measure the bisphenol A solution. We eluted bisphenol A with mobile phase methanol and water (55:45 by volume). The degradation rate of BPA was calculated using the following Equation (1):

(1) $$\eta =\frac{C_{0}-C_{t}}{C_{0}}\times 100$$

where *C_t_* is the concentration of BPA at time *t* and *C*_0_ is the initial concentration of BPA before the reaction.

## 3. Result and Discussion

### 3.1. Photocatalytic Performances Test

**Figure 1:**
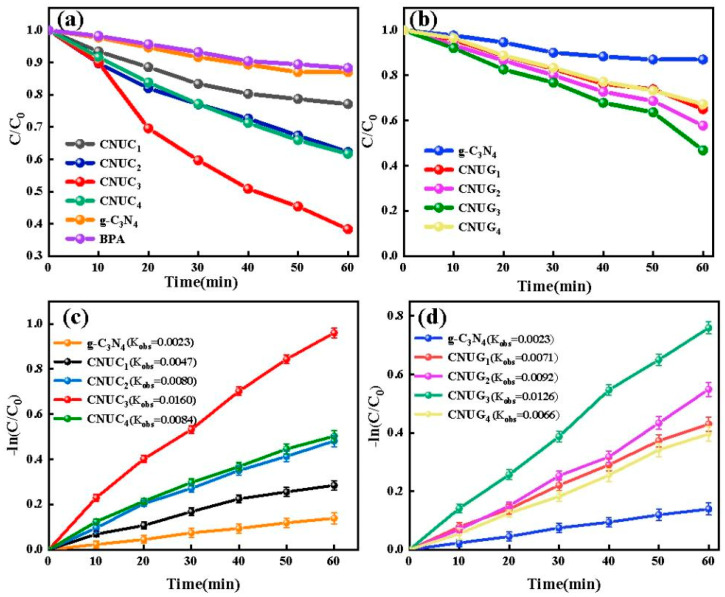
Photocatalytic performance test results: (**a**,**b**) Photocatalytic degradation of BPA over pure g-C_3_N_4_, CNUC, and CNUG samples under visible light irradiation. (**c**,**d**) Corresponding pseudo-first-order reaction kinetic fitted curves of g-C_3_N_4_, CNUC and CNUG samples.

Under the irradiation of visible light, using the prepared catalyst as a probe and BPA as the target pollutant, the photocatalytic properties of pure g-C_3_N_4_, CNUC and CNUG were explored. As shown in Figure 1a, the degradation rate of BPA with pure g-C_3_N_4_ as a photocatalyst was only 14% after a reaction of 60 min, indicating that only 14% BPA was removed. Compared with pure g-C_3_N_4_, the four CNUC_1–4_ samples presented higher photocatalytic efficiency for BPA degradation. The degradation rate of pure BPA is 11%. The degradation rate of BPA with CNUC_1_, CNUC_2_, CNUC_3_, and CNUC_4_ as photocatalysts was 48%, 52%, 81%, and 53%, respectively, corresponding to 3, 4, 7, and 4 times higher rates than that of BPA with pure g-C_3_N_4_, as a photocatalyst. The photodegradation performance was observed to increase with the increase in (NH_4_)_2_C_2_O_4_ loading from 1 to 3 mmol and deteriorated with a further increase in (NH_4_)_2_C_2_O_4_ loading to 4 mmol, probably due to the blocking effect of light absorption when a higher amount of (NH_4_)_2_C_2_O_4_ was incorporated. Similar phenomena were observed for CNUG samples, as shown in Figure 1b. The degradation rate of BPA with g-C_3_N_4_, CNUG_1_, CNUG_2_, CNUG_3_, and CNUG_4_ as photocatalysts was 35%, 62%, 72%, and 42%, respectively, corresponding to a 2.5, 4, 5, and 3 times higher rate than that of BPA with pure g-C_3_N_4_ as a photocatalyst. Among all of the CNUC and CNUG samples, CNUC_3_ presented the best performance. The photocatalytic degradation kinetics of BPA could be well expressed by pseudo-first-order reaction using the following equation:

(2) $$-\ln(C_{t}/C_{0})=kt$$

where *C_t_* is the concentration of BPA at a specific time *t*, *C*_0_ is the original concentration, and *k* is the rate constant. As shown in Figure 1c, these BPA photodegradation reactions followed first-order kinetics closely. The value of k was calculated to be 0.0125, 0.0165, 0.0278, and 0.0183 min^−1^ for BPA with CNUC_1_, CNUC_2_, CNUC_3_, and CNUC_4_ as photocatalysts, respectively, corresponding to 5, 7, 12, and 8 times faster than that of BPA with pure g-C_3_N_4_ as a photocatalyst. Figure 1d presents the pseudo-first-order fitting for BPA with CNUG as a photocatalyst. The obtained rate constant for CNUG_1_, CNUG_2_ CNUG_3_, and CNUG_4_ was 0.0090, 0.0091, 0.0207, and 0.0072 min^−1^, respectively. The highest rate constant observed for BPA with CNUG_3_ as a photocatalyst was 9 times higher than that for BPA with pure g-C_3_N_4_ as a photocatalyst.

### 3.2. Characterization of Photocatalysts

#### 3.2.1. Catalysis Structure and Morphology Analysis 

**Figure 2:**
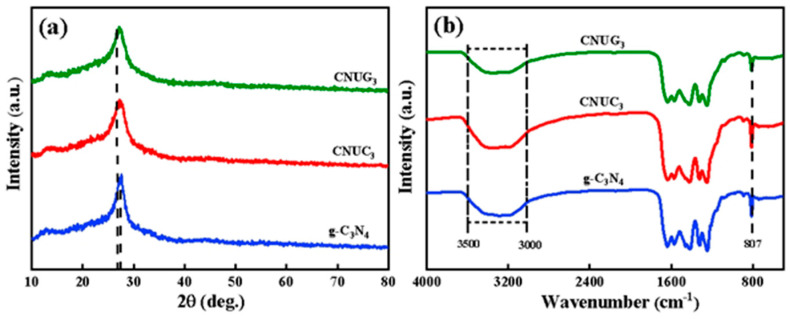
(**a**) XRD patterns; (**b**) FT-IR spectra of g-C_3_N_4_, CNUC_3_, and CNUG_3_.

The phase composition and the crystal structure of g-C_3_N_4_, CNUC_3_, and CNUG_3_ were characterized by XRD, as shown in Figure 2a. All samples revealed a typical g-C_3_N_4_ layered structure, and the result show the presence of two diffraction peaks at 13.0° and 27.5°, which were attributed to the (100) and (002) crystal planes, respectively [24]. The diffraction peak at 2*θ* = 27.5° was attributed to the interlayer stacking of aromatic compounds, and the diffraction peak at 2*θ* = 13.0° was derived from the in-plane stacking of the tris–s–triazine ring. [25]. Compared with that of g-C_3_N_4_, the (002) diffraction peak of CNUC_3_ and CNUG_3_ was shifted from 27.61° to 27.31°, indicating that the interplanar stacking distance increased after O-doping.

Figure 2b shows the FT-IR spectra of g-C_3_N_4_, CNUC_3_, and CNUG_3_. All of the samples revealed similar FT-IR patterns. g-C_3_N_4_ had an obvious characteristic peak at about 809 cm^−1^, which was assigned to the tri-s-triazine ring. The multiple diffraction peaks within 1200 to 1700 cm^−1^ represented the stretching vibration of the C–N heterocyclic ring [26], and the broad peak between 3000 to 3500 cm^−1^ was caused by the stretching vibration of N–H and the water molecules adsorbed on the catalyst surface. [27]. Figure 2b shows that the enhanced peak intensity of CNUC_3_ at between 3000 and 3500 cm^−1^ indicated the presence of more −OH bonds on the surface of CNUC_3_.

**Figure 3:**
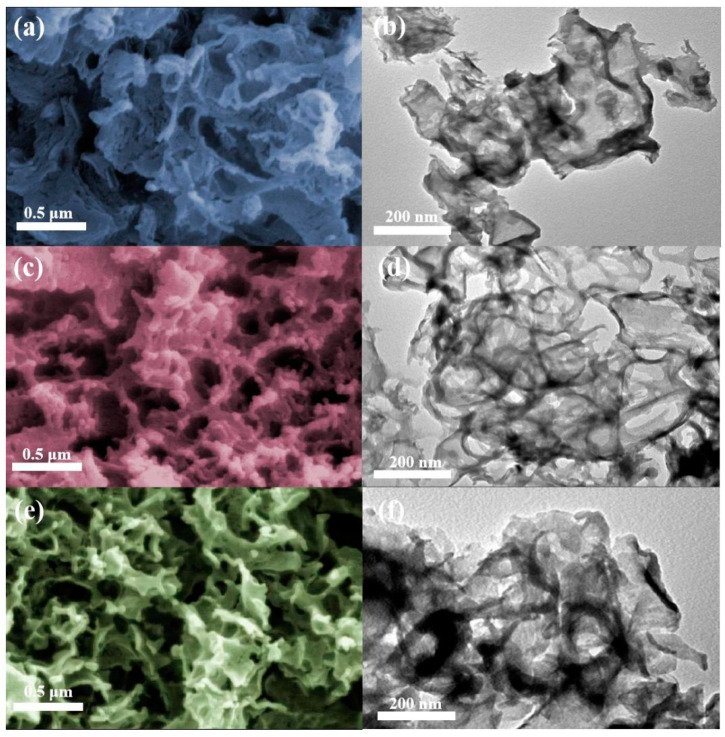
Morphology analysis test results: SEM images of (**a**) g-C_3_N_4_, (**b**) CNUC_3_, and (**c**) CNUG_3_. TEM images of (**d**) g-C_3_N_4_, (**e**) CNUC_3_, and (**f**) CNUG_3_.

The morphologies of the g-C_3_N_4_, CNUC_3_, and CNUG_3_ were characterized by SEM and TEM. As shown in Figure 3a,d,e, pure g-C_3_N_4_ showed large bulk particles, a smooth surface and a thick 2D sheet-like structure, while CNUC_3_ had a porous structure with a skeleton shape, owing to the release of gases (NH_3_, H_2_O, CO, and CO_2_) due to the decomposition of (NH_4_)_2_C_2_O_4_ during the pyrolysis process [28]. CNUG_3_ showed obvious flake-like structures with some disordered pores, which might have resulted from the evolution of carbonaceous gas from glycine oxidation at high temperatures [29]. Compared with CNUC_3_, CNUG_3_ had fewer holes. In addition, the porous structure provided the CNUC_3_ and CNUG_3_ catalysts with a higher specific surface area and a larger pore volume, thereby exposing more active sites and resulting in enhanced photocatalytic activity. The TEM image showed that the as-prepared CNUC_3_ samples consisted of the ultrathin 2D layers with abundant mesoporous layers compared to g-C_3_N_4_ and CNUG_3_ with a compact microstructure. From the morphological point of view, consistent with the TEM image, the pore structure of CNUG_3_ was not as good as that of CNUC_3_. The knitting of ultrathin and surface porous CNUC_3_ nanosheets could shorten the distance between the body and the surface diffuse migration of photo-generated charges, thus inhibiting the photogenerated charges in the internal recombination. Figures S1 and S2 show the energy-dispersive X-ray analysis maps of the CNUC_3_ and CNUG_3_ sample. The C, N, and O elements were uniformly dispersed on the CNUC_3_ and CNUG_3_ photocatalysts.

The N_2_ adsorption–desorption isotherms of the as-prepared samples are shown in Figure S3. The isotherm of the sample was type IV and the hysteresis loop was type H3 [30]. As displayed in Table S1, compared with g-C_3_N_4_ and CNUG_3_, CNUC_3_ showed higher pore volumes, which was consistent with the result of TEM. Furthermore, the surface area (S_BET_) of CNUC_3_ was 48.28 m^2^g^−1^, which was higher than that of CNUG_3_ (45.27 m^2^g^−1^) and much higher than that of g-C_3_N_4_ (14.93 m^2^g^−1^). The increase in the surface area of the CNUC_3_ sample was conducive to photocatalytic performance. According to the existing literature, a larger area could increase the number of adsorption and photocatalytic reaction sites, which was conducive to photocatalytic reactions because it enhanced mass transfer and light collection efficiency.

**Figure 4:**
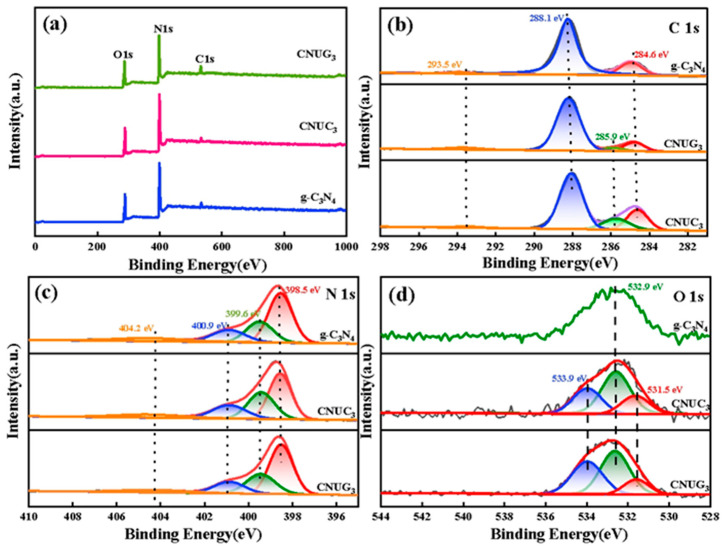
Elemental compositions and the chemical binding states of the photocatalyst: (**a**) Survey spectrum of g-C_3_N_4_ CNUC_3_, and CNUG_3_. High-resolution XPS spectra of (**b**) C 1s, (**c**) N 1s, and (**d**) O 1s for g-C_3_N_4_ CNUC_3_ and CNUG_3_ samples.

The elemental compositions and the chemical binding states of the photocatalyst composites were analyzed by XPS. The XPS spectra of g-C_3_N_4_, CNUC_3_, and CNUG_3_ are presented in Figure 4a. In Figure 4b, it could be seen that the C 1s XPS spectrum of g-C_3_N_4_ was mainly divided into three characteristic peaks at 284.60, 288.1 and 293.5 eV. The binding energy at 284.6 eV was indefinite carbon (C–C/C=C) [31]. The characteristic peak at 288.1 eV was attributed to the sp^2^ bonding carbon in the s-triazine ring (N–C=N), and this binding energy was the main carbon characteristic peak of g-C_3_N_4_ [32]. The peak at 293.5 eV could be assigned to π electronic excitation [33]. The new peaks at 285.9 eV confirmed the generation of C–O bonds in CNUC_3_ and CNUG_3_ samples [34]. The peak area of the C–O bond was noted to be the largest for CNUC_3_, indicating that the amount of oxygen doped in CNUC_3_ was the highest. The N 1s spectrum of the sample was divided into four characteristic peaks located at 398.5, 399.6, 400.9 and 404.2 eV. The characteristic peak of 398.5 eV was the pyridine nitrogen of the triazine ring in C=N–C. The peak with binding energy at 399.6 eV corresponded to the bridging N atom in tertiary nitrogen (N–(C)_3_). The binding energy was at 400.9 and 404.2 eV. It is attributed to the presence of nitrogen atoms in the amino group (C–N–H) and π excitation, respectively [35]. The peak area of N 1s peak at 398.5 eV attributed to the C=N–C group for CNUG_3_ was reduced compared with that of pure g-C_3_N_4_ and CNUG_3_. This might be due to the sp^2^ hybridized triazine structure introduced into the N-containing aromatic ring in CNUC_3_, which caused the N atom to be replaced by the multi-electron O atom and gradually destroyed. In the spectrum of O 1s in Figure 4d, the new peaks at 531.5 eV for CNUC_3_ and CNUG_3_ might be attributed to the formation of O–C species in the crystal lattice by O doping [36]. The peak area at 531.5 eV for CNUC_3_ was higher than that of CNUG_3_, indicating that the O atom in ammonium oxalate easily replaced the N atom in the g-C_3_N_4_ structure to form C–O.

#### 3.2.2. Optical and Electrochemical Properties

**Figure 5:**
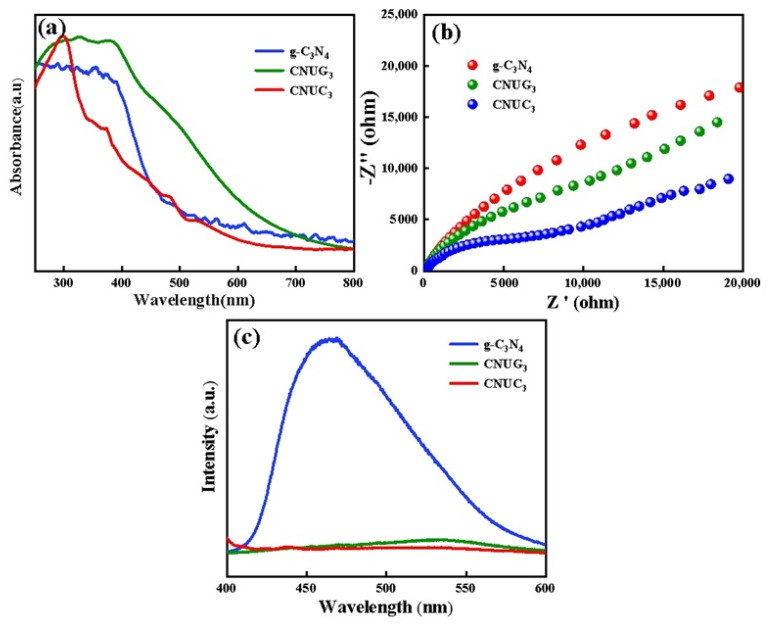
Electrochemical characterization: (**a**) UV–vis absorption spectra; (**b**) EIS Nyquist plots; (**c**) steady-state PL spectra of g-C_3_N_4_ CNUC_3_ and CNUG_3_.

To confirm the light absorption and electronic structure of the catalysts, UV-vis spectroscopy was performed on g-C_3_N_4_, CNUC_3_, and CNUG_3_. From the UV-vis spectra of g-C_3_N_4_, CNUC_3_, and CNUG_3_ shown in Figure 5a, we could see that all samples showed strong absorption in the visible light region. The absorption edges of g-C_3_N_4_ were located at around 489 nm. Both CNUC_3_ and CNUG_3_ samples exhibited a redshifted in the absorption edges at 696 and 650 nm, respectively. These results indicate that CNUC_3_ and CNUG_3_ could absorb a wider spectrum and more light photon energy, leading to an increase in the production of electrons and holes. Compared with CNUG_3_, CNUC_3_ showed a wider spectrum range, indicating a possibly improved visible light photocatalytic activity. The corresponding optical bandgap energy (*Eg*) was calculated using the following formula:

(3) $$\alpha hv=A{\left(hv-E_{g}\right)}^{n/2}$$

where *α*, *hν*, *A*, and *Eg* represent the absorption coefficient, light energy, constant, and bandgap, respectively. The bandgap of g-C_3_N_4_, CNUC_3_, and CNUG_3_ was determined to be 2.77, 2.65, and 2.45 eV, respectively, as shown in Figure S4. Chen [18], 1.5% SO-g-C_3_N_4_, had a corresponding band gap of 2.63 eV. Compared with Chen, the CNUC_3_ and CNUG_3_ prepared in this paper significantly reduced the forbidden band width of the catalyst, and the wavelength shifts to the visible light direction, so that the catalyst could make better use of visible light. The narrow band gap could enable the catalyst to respond to visible light, shift the wavelength to the visible light direction, and the catalyst could capture more visible light photons, which helped to improve the photocatalytic performance of the catalyst.

The separation efficiency and the transferability of photoexcited electrons and holes were evaluated using the photoluminescence (PL) emission response and EIS. Figure 5b presents the EIS spectra of g-C_3_N_4_, CNUC_3_, and CNUG_3_. The smaller arc radius in the EIS Nyquist diagram, illustrated a stronger photogenerated charge separation and transferability. It could be seen from the EIS Nyquist diagram that CNUC_3_ had the smallest arc radius, followed by CNUG_3_, and g-C_3_N_4_ had the largest arc radius. This result shows that CNUC_3_ had a good photo-generated carrier separation ability and further confirms that CNUC_3_ had good photocatalytic activity, which was consistent with the results discussed previously. Figure 5c presents the PL spectra of g-C_3_N_4_, CNUC_3_, and CNUG_3_. Generally, the stronger PL intensity indicated the faster combination of photogenerated charge. As shown in the steady-state PL spectra, g-C_3_N_4_ depicted an obvious PL peak with a strong emission intensity. The incorporation of either (NH_4_)_2_C_2_O_4_ or C_2_H_5_NO_2_ led to a significant reduction in the intensity of the PL peaks for CNUC_3_ and CNUG_3_, indicating the efficient separation of light-induced electron–hole pairs. Since the PL intensity was mainly derived from the recombination of photogenerated carriers in the photocatalyst, the reduction in PL intensity was crucial evidence for increasing the charge separation rate. CNUC_3_ showed the lowest fluorescence intensity compared with the other two samples, which was consistent with the EIS spectra. Meanwhile, the emission peaks were red-shifted from 465 to 539 nm due to the O doping, which could promote the π-electron delocalization of the g-C_3_N_4_ network.

**Figure 6:**
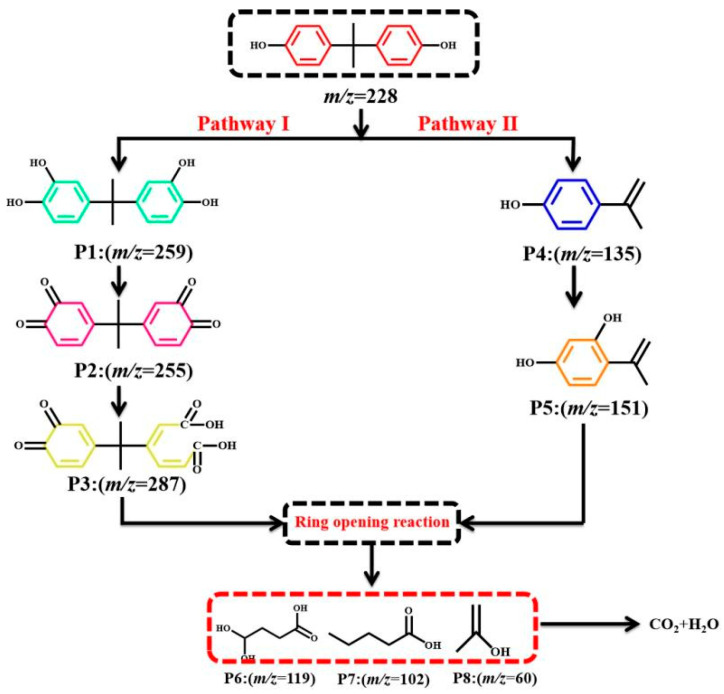
Possible degradation pathway of BPA under CNUC_3_ system.

In order to explore the intermediates and possible degradation pathways generated by BPA in the photocatalytic degradation process, LC−MS was used to analyze the mineralization evolution process of the catalyst degradation of BPA. Similar work had been carried out in the author’s previous research [22]. In Figure 6, two possible BPA degradation pathways were proposed [37,38]. According to reports in the literature, the current active oxidative free radicals degrade BPA molecules through two possible mechanisms: C−C bond cleavage and hydroxylation. In the first approach, the benzene ring in BPA undergoes hydroxylation reaction to form dihydroxylated BPA (wt: 259), and the OH group on the dihydroxylated BPA molecule could be further oxidized to form tetracarbonylation BPA (wt: 255), and tetracarbonylation BPA was further oxidized to produce [2E,4Z)-3-(2-(3,4-dioxane)hexane-1,5-ethylene-1-yl)] hexane-2,4-dienedioic acid (wt: 287). In the second way, the C−C bond connected to the benzene ring in BPA reacts with the active material to form 4-isopropylphenol (wt: 135), and 4-isopropylphenol was further oxidized to form 1-(2,4-dihydroxy-yphenyl) ethanone (wt: 151). Finally, all of the above aromatic compounds undergo ring-opening reactions to produce small molecular compounds, such as 1-dihydroxybutyric acid (wt: 119), valeric acid (wt: 102), and acetic acid (wt: 60), etc. These small molecular compounds with further mineralization generate CO_2_ and H_2_O.

#### 3.2.3. Mechanisms of Enhanced Photocatalytic Performance

**Figure 7:**
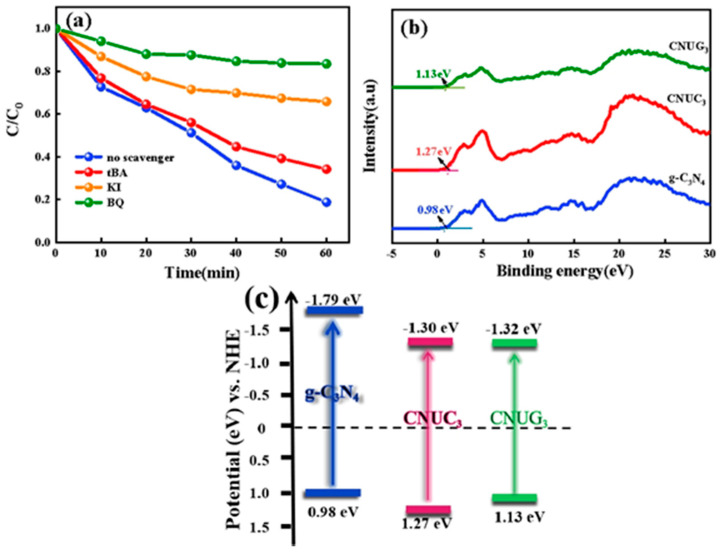
Mechanism discussion: (**a**) effect of different radical scavengers on the photodegradation of BPA over CNUC_3_; (**b**) XPS VB spectra; (**c**) schematic illustration of the energy-level diagrams of the as-obtained catalysts.

The photocatalytic mechanism of the degradation of organic contaminants in CNUC_3_ samples under visible light irradiation was explored by free radical trapping experiments. tert-Butyl alcohol (t-BA), p-benzoquinone (BQ), and KI were taken as the quenchers for •OH, •O_2_^−^, and h^+^, respectively. It could be seen from Figure 7a that t-BA could slightly inhibit the degradation of BPA, and BQ and KI had a significant inhibitory effect on BPA, reducing BPA by 66% and 47.8%, respectively. The above results show that •O_2_^−^ and h^+^ were the main active substances in the degradation of BPA and played a major role in photocatalytic degradation. The bandgaps of g-C_3_N_4_, CNUC_3_, and CNUG_3_ were discussed earlier by UV-vis diffuse reflectance spectra. The bandgap of solid solutions of g-C_3_N_4_, CNUC_3_, and CNUG_3_ was 2.77, 2.45, and 2.65 eV, respectively. The XPS valence band spectra of g-C_3_N_4_, CNUC_3_ and CNUG_3_ are shown in Figure 7b. The valence band (VB) spectra for g-C_3_N_4_, CNUC_3_, and CNUG_3_ were 0.98, 1.27, and 1.13 eV, respectively, and the corresponding conduction band (CB) calculated from the equation E_CB_ = E_VB_ − E_g_, was −1.79, −1.18, and −1.52 eV, respectively. The band structures of g-C_3_N_4_, CNUC_3_, and CNUG_3_ are presented in Figure 7c. According to previous literature reported [39], the N1 and N4 sites in g-C_3_N_4_ were replaced by oxygen atom in CNUC_3_ catalyst prepared by ammonium oxalate as precursor, while the N1 site in g-C_3_N_4_ was replaced by glycine as precursor [29]. Therefore, CNUC_3_ has a narrower band gap, which improves the visible light capture ability and photocatalytic activity.

**Figure 8:**
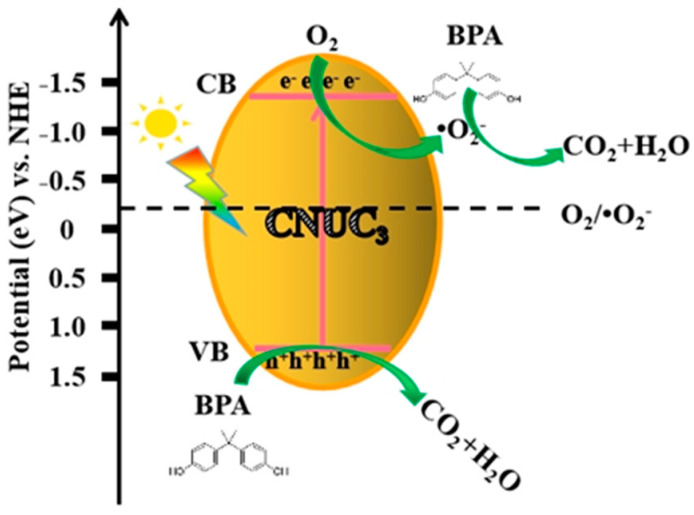
Schematic diagram of the CNUC_3_ under visible light irradiation.

Based on the above experiment and analysis, a possible mechanism for the photodegradation of BPA by CNUC_3_ under visible light irradiation was proposed, as shown in Figure 8. First, under the irradiation of visible light, photogenerated e^−^ transferred from the VB to the CB of CNUC_3_ and formed a conjugated delocalized π system, because the potential on CNUC_3_ CB (−1.18 to −1.79 eV) was more negative than the O_2_/•O_2_^−^ (−0.33 eV vs. NHE). The e^−^ on CB could directly react with O_2_ on the surface of the catalyst to produce •O_2_^−^. Due to the strong oxidation ability of •O_2_^−^ radicals, it could directly mineralize BPA into CO_2_ and H_2_O. Besides, the VB potential of CNUC_3_ was lower than the standard oxidation-reduction potential of OH^−^/•OH (+2.38 eV vs. NHE) [27], so that h^+^ left on the VB of CNUC_3_ could not oxidize OH^−^ to produce •OH kinetically. However, the h^+^ on CNUC_3_ VB could directly react with BPA to produce CO_2_ and H_2_O. On the one hand, O atom doping was introduced and porous structure built an inter-bandgap, leading to a narrow bandgap, which could increase the visible light absorption and light quantum efficiencies and further enhanced the photocatalytic activity under visible light. On the other hand, O-doped g-C_3_N_4_ could effectively improve the separation of photo-generated carriers and accelerated the improvement of charge transfer efficiency, thereby enhancing its photocatalytic activity.

**Figure 9:**
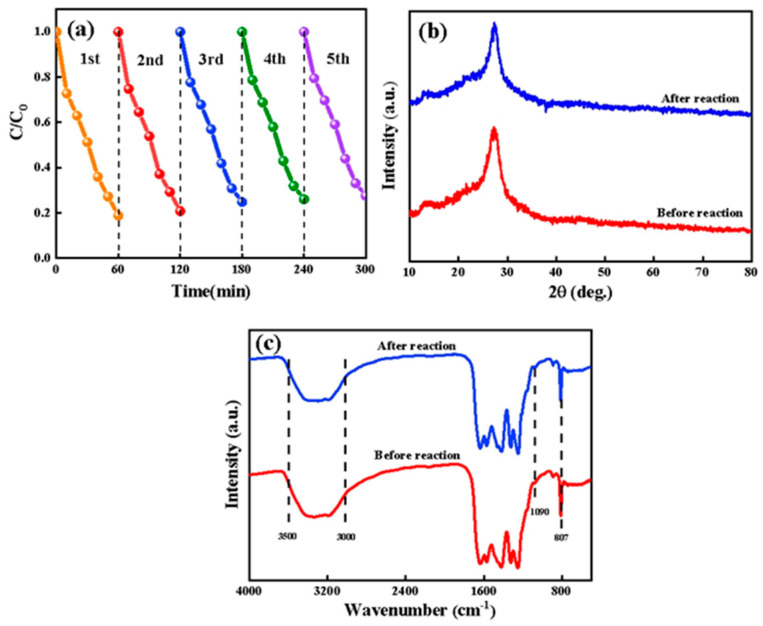
Catalyst stability research: (**a**) Reusability evaluation of CNUC_3_ for the photodegradation of BPA under visible light; (**b**) XRD patterns; (**c**) FT-IR spectra of the CNUC_3_ sample before and after five photocatalytic reaction cycles.

In addition to investigating the photocatalytic activity of the catalyst, the reusability of the photocatalyst was also the key to its industrialization. Five photocatalytic cycle experiments were carried out on CNUC_3_ to evaluate its light stability and reusability. Figure 9a was a photocatalytic cycle experiment diagram. It could be seen from the figure that after five cycles of the catalyst, the degradation efficiency of BPA was 75%. During the reaction process, a small amount of CNUC_3_ powder would be removed due to the continuous stirring process, and a small amount of catalyst would also be removed during the washing and drying process of the catalyst. It could be seen from the figure that after the photocatalytic reaction was cycled three times, the catalyst activity tends to be stable. This phenomenon showed that CNUC_3_ had good stability and reusability. In addition, in order to explore whether the crystal form and chemical species of the catalyst changed after five cycles, the CNUC_3_ was characterized by XRD and FT-IR. Figure 9b was the XRD pattern after five cycles. From the figure, it could be seen that after five cycles of the catalyst, all crystal faces hardly changed. Figure 9c was the FT-IR diagram after five cycles. The results show that the characteristic peaks of the FT-IR spectrum before and after the reaction hardly changed. From the above results, CNUC_3_ had good chemical stability and can effectively inhibit photo-corrosion performance.

## 4. Conclusions

In summary, we successfully fabricated O-doped porous g-C_3_N_4_ nanosheet photocatalysts via thermal copolymerization with urea and ammonium oxalate or glycine as raw materials. A large number of pores were formed by gas generated from the copolymerization decomposition of ammonium oxalate, resulting in an enlarged specific surface area. At the same time, O doping adjusted the g-C_3_N_4_ bandgap engineering to narrow the bandgap and expand the visible light response.

(1) Under visible light irradiation, the optimum photocatalytic efficiency of CNUC_3_ and CNUG_3_ nanosheets was enhanced by nearly seven and five times, respectively, more than that of g-C_3_N_4_ for BPA degradation in 60 min. CNUC_3_ was found to present high stability and excellent recycling in the photocatalytic degradation processes.
(2) Free radical capture experiments have confirmed that h^+^ and •O_2_^−^ were the main active species that degrade BPA, and •O_2_^−^ could reduce BPA to produce CO_2_ and H_2_O. Meanwhile, h^+^ had strong oxidizing ability and could directly mineralize BPA into small molecular substances.
(3) Therefore, this work may open a new route to fabricate nonmetal-modified g-C_3_N_4_-based catalysts with high degradation efficiency under visible light irradiation. Moreover, it may provide new inspiration for improving the activities of photocatalysts.

## Data Availability

The data presented in this study are available upon request from the corresponding author.

## Supplementary Materials

The following supplementary materials can be downloaded at: https://www.mdpi.com/article/10.3390/ma15041391/s1, Figure S1: Mapping images of CNUC3; Figure S2: Mapping images of CNUG3; Figure S3: N2 adsorption–desorption isotherms of g-C3N4 CNUC3 and CNUG3; Table S1: Textural properties of the prepared samples; Figure S4: Bandgap value, estimated using a related curve of (αhν)^1/2^ versus photon energy.
