# Supplementary material for: Porous Oxygen-Doped g-C3N4 with the Different Precursors for Excellent Photocatalytic Activities under Visible Light
Source: Materials (Basel). 2022 Feb 14;15(4):1391. doi: 10.3390/ma15041391 (PMC8877032; doi:10.3390/ma15041391)
Supplement: Supplementary file 1 [file materials-15-01391-s001.zip › materials-1499200-supplementary.pdf]

materials

MDPI

Supplementary Material

Porous Oxygen-Doped g-C_3N_4 with the Different Precursors for Excellent Photocatalytic Activities under Visible Light

Jiajing Zhang, Yongjie Zheng *, Heshan Zheng, Tao Jing, Yunpeng Zhao and Jingzhi Tian *

School of Chemistry and Chemical Engineering, Qiqihar University, Qiqihar 161006, China; zhangjiajing_1994@163.com (J.Z.); zhengheshan001@163.com (H.Z.); jtkr@163.com (T.J.); zhyypp@163.com (Y.Z.)

* Correspondence: zyj1964@163.com (Y.Z.); tjz6666@163.com (J.T.); Tel: +86-0452-2738-152 (Y.Z.)

2. Experimental section

2.2. Preparation

Synthesis of g-C_3N_4: Two-dimensional (2D) g-C_3N_4 nanosheets were prepared using a thermal polymerization method. Specifically, 10 g of urea was first heated up to 550 °C and the heating rate of 5 °C/min and kept at this temperature 2 h. Subsequently, the ob-tained yellow bulk product was collected after being cooled down to room temperature and milled into powders.

Synthesis of CNUC and CNUG: Typically, 1, 2, 3, and 4 mmol of $(NH_4)_2C_2O_4$ were added into 15 mL of aqueous suspensions containing urea (0.17mol) and then ultrasonicated for 30 min, followed by vigorous stirring for 2 h before being dried at 80 °C to remove the solvent. After drying, the white solids were put into a 50-mL ceramic crucible with a lid, then placed in a muffle furnace and heated up to 550 °C at a heating rate of 5 °C/min, and subsequently kept at that temperature for 2 h. Finally, the resultant products were obtained and denoted as $CNUC_X$ (X = 1, 2, 3, 4) where X represents the mass of $(NH_4)_2C_2O_4$. The CNUG sample was prepared using $C_2H_5NO_2$ instead of $(NH_4)_2C_2O_4$ following the aforementioned procedures; the synthesized samples were denoted as $CNUG_X$ (X = 1, 2, 3, 4).

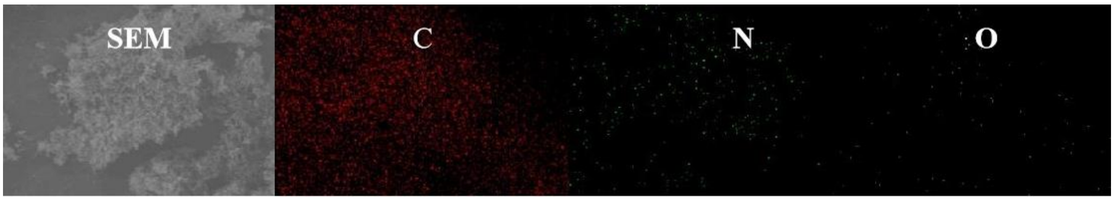

Figure S1. Mapping images of $CNUC_3$.

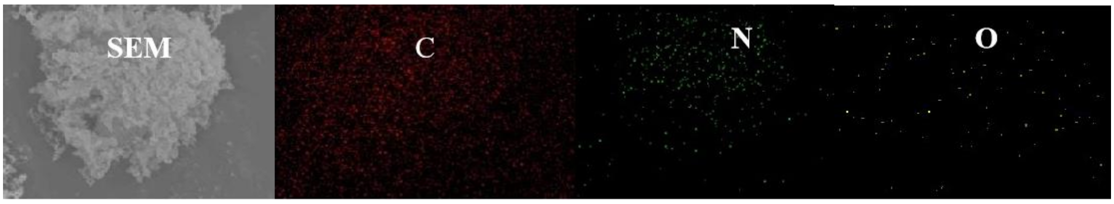

Figure S2. Mapping images of $CNUG_3$.

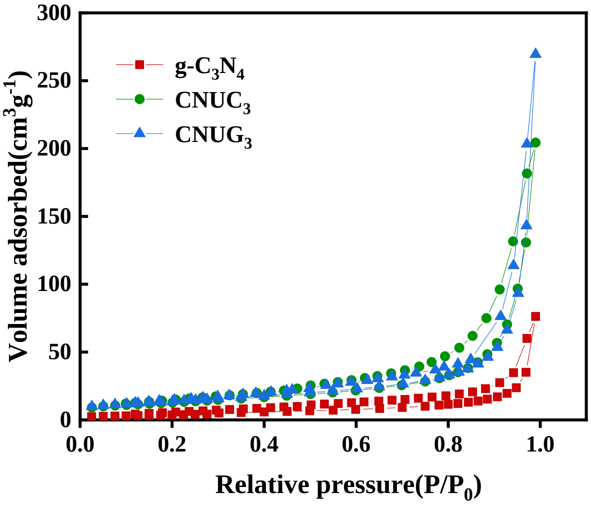

Figure S3. N_2 adsorption–desorption isotherms of g-C_3N_4 $CNUC_3$ and $CNUG_3$.

Table S1. Textural properties of the prepared samples.

Samples	S_{BET} (m^2g^{-1})	Pore size (nm)
g-C_3N_4	14.94	0.092
$CNUC_3$	48.28	0.313
$CNUG_3$	45.27	0.285

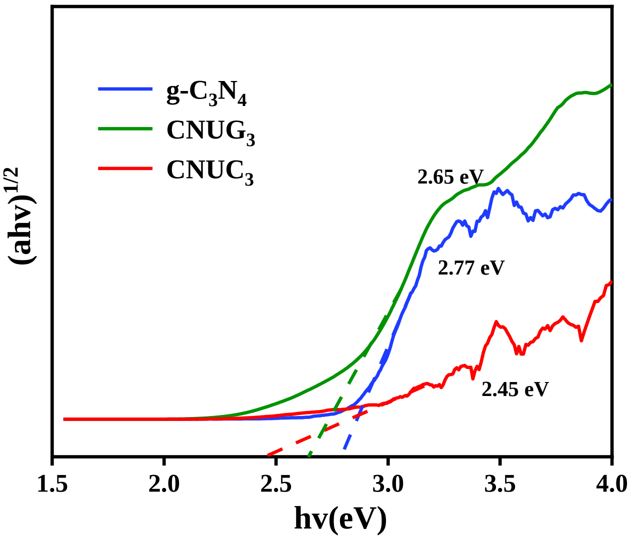

Figure S4. Bandgap value, estimated using a related curve of $(\alpha h\nu)^{1/2}$ versus photon energy.
